# Molecular Profiling of Cutaneous Lupus Lesions Identifies Subgroups Distinct from Clinical Phenotypes

**DOI:** 10.3390/jcm8081244

**Published:** 2019-08-17

**Authors:** Celine C. Berthier, Lam C. Tsoi, Tamra J. Reed, Jasmine N. Stannard, Emily M. Myers, Rajaie Namas, Xianying Xing, Stephanie Lazar, Lori Lowe, Matthias Kretzler, Johann E. Gudjonsson, J. Michelle Kahlenberg

**Affiliations:** 1 Division of Nephrology, Department of Internal Medicine, University of Michigan, Ann Arbor, MI 48109, USA; 2 Department of Dermatology, Department of Computational Medicine & Bioinformatics, Department of Biostatistics, University of Michigan, Ann Arbor, MI 48109, USA; 3 Division of Rheumatology, Department of Internal medicine, University of Michigan, Ann Arbor, MI 48109, USA; 4 IHA Rheumatology Consultants, Ann Arbor, MI 48109, USA; 5 Lifebridge Health, Baltimore, MD 21215, USA; 6 Division of Rheumatology, Department of Internal Medicine, Cleveland Clinic Abu Dhabi, 112412 Abu Dhabi, United Arab Emirates; 7 Department of Dermatology, University of Michigan, Ann Arbor, MI 48109, USA; 8 Department of Pathology, University of Michigan, Ann Arbor, MI 48109, USA

**Keywords:** systemic lupus erythematosus, interferon, cutaneous lupus, discoid

## Abstract

Cutaneous lupus erythematosus (CLE) is a common manifestation of systemic lupus erythematosus (SLE), and CLE can also develop without systemic involvement. CLE can be difficult to treat and negatively contributes to quality of life. Despite the importance of CLE, our knowledge of what differentiates cutaneous lupus subtypes is limited. Here, we utilized a large cohort of 90 CLE lesional biopsies to compare discoid lupus erythematosus (DLE) and subacute cutaneous lupus (SCLE) in patients with and without associated SLE in order to discern the drivers of disease activity and possibly uncover better treatment targets. Overall, we found that DLE and SCLE share many differentially expressed genes (DEG) reflecting type I interferon (IFN) signaling and repression of EGFR pathways. No differences between CLE only and SLE-associated CLE lesions were found. Of note, DLE uniquely expresses an IFN-γ node. Unbiased cluster analysis of the DEGs identified two groups separated by neutrophilic vs. monocytic signatures that did not sort the patients based on clinical phenotype or disease activity. This suggests that unbiased analysis of the pathobiology of CLE lesions may be important for personalized medicine and targeted therapeutic decision making.

## 1. Introduction

Cutaneous lupus erythematosus (CLE) is a disfiguring disease that can exist as an independent entity or as a manifestation of systemic lupus erythematosus (SLE) where up to 70% of patients experience lesions during their disease course [1]. Despite differing clinical presentations, some characteristics, such as increased IL-18 receptor expression and type I interferon (IFN) gene upregulation, have been histologically and transcriptionally described to be similar in DLE and SCLE [1,2,3,4]. Subacute CLE (SCLE) is an inflammatory lesion with associated erythema in papulosquamous or annular formations. Typically, SCLE does not scar but depigmentation can occur [5]. Discoid lesions (DLE) are often circular and frequently lead to alopecia and scar formation [5]. SCLE lesions have a higher propensity for photo-provocation [6] and a more robust inflammatory infiltrate following ultraviolet B (UV) exposure [7]. The pathogenic mechanisms which govern the differences between DLE and SCLE remain poorly defined, and this is reflected by the refractory nature of cutaneous lesions to usual lupus therapies and no clear treatment guidelines that differentiate the subtypes [8].

Comparison of lesional to non-lesional skin in six cutaneous-only DLE patients revealed a strong interferon signature and apoptosis signaling, but two of the six patients were on topical steroids, which may have altered the gene expression signature [9]. Another study which compared seven DLE biopsies to psoriasis revealed a stronger IFN-regulated gene presence and a lower Th17 profile in DLE compared to psoriasis [10]. Others have looked at small panels of cytokine and chemokine pathways which have shown reduced expression of IFN-ɣ and TNF-α in DLE when compared to SCLE, but this has not been put in clinical context or in perspective to control skin [3].Thus, a great need exists to develop a comprehensive understanding of the regulation of inflammatory pathways in cutaneous lupus that will provide targets for additional mechanistic studies, give insight into the pathogenic differences in lesion subtype, and allow for tailoring of treatments to individual patients based on rash phenotypes/expression.

In this study, we evaluated the transcriptional profiles of 47 DLE and 43 SCLE biopsies and compared them to control skin and to each other in order to develop a comprehensive understanding of the similarities and differences between these two CLE subtypes. Ultimately, we found that transcriptional changes dictated subgroups that separate based on inflammatory composition and epidermal gene expression rather than DLE vs. SCLE subgroupings.

## 2. Materials and Methods

### 2.1. Sample Acquisition

Cases of DLE and SCLE biopsies were identified via a SNOMED search of the University of Michigan Pathology Database using the search terms “lupus” and “cutaneous lupus” under IRBMED #HUM72843. Patients who met both clinical and histologic criteria for DLE or SCLE were included in the study. An attempt was made to exclude patients on topical steroids to avoid a strong glucocorticoid signature. Patient biopsies were from various locations based on the site of active lesions. Predominant locations included upper arm, neck, upper back, face, and scalp. Control biopsies were primarily from non-sun-exposed upper thigh. Disease activity scores for SLE Disease Activity Index (SLEDAI) and cutaneous lupus erythematosus disease area and severity index (CLASI [11]) at the time of biopsy were calculated via chart review. See Table 1 for demographic information.

### 2.2. RNA Isolation and Microarray Procedures

Formalin-fixed paraffin embedded (FFPE) blocks of diagnostic CLE skin biopsies were obtained from the University of Michigan pathology archive and from Kahlenberg and Gudjonsson labs for controls, and five 10 μm sections were cut with a microtome. RNA was extracted using the E.N.Z.A. FFPE RNA Kit (Omega Bio-tek) following the manufacturer’s instructions. RNA was quantified via Nanodrop. Complementary DNA (cDNA) was prepared (NuGEN, Ovation PicoSL WTA System V2 Manual, P/N M01226 v4) from approximately 30 ng total RNA and 2.5 ug cDNA was biotinylated using the NuGEN Encore Biotin Module (Encore Biotin Module Manual, P/N M01111 v6). The Poly- A RNA Control Kit was used as the routine procedure at the University of Michigan (U-M) microarray core. Labeled cDNA was hybridized at 48 °C to Affymetrix Human Gene ST 2.1 array plates, which were then washed, stained and scanned using the Affymetrix GeneTitan system (software version 3.2.4.1515) with the assistance of the U-M DNA Sequencing Core. Quality control and RMA (Robust Multi-array Average) [12] normalization of CEL files were performed in R software version 3.1.3 using custom CDF version 19 and modified Affymetrix_1.44.1 package from BrainArray [13]. Log_2_ expression values were batch corrected using Combat implemented into GenePattern [14]. The baseline expression was defined as minimum plus one standard deviation of the median of all genes. A variance filter of 80% was then applied. Of the 25,582 unique genes represented on the Human ST2.1 chip, a total of 20,410 genes passed the defined criteria. Data from CLE microarrays are available through GEO GSE81071.

### 2.3. Calculation of IFN Scores

IFN scores were calculated using 6 IFN genes (*IFIT1*, *IRF7*, *MX1*, *EIF2AK2*, *OASL*, *IFI44L*) using the algorithm described by Feng et al. [15].

### 2.4. Literature-Based Network and Pathway Analyses, Hierarchical Clustering, Heatmap Generation, and Gene List Comparison

Significantly regulated genes were analyzed by creating biological literature-based networks using Genomatix Pathway System (GePS version 2.10.1, Munich, Germany) [16] and the function-word level as minimum evidence level parameter. Canonical pathways were identified using Ingenuity Pathway Analysis software (IPA) (www.ingenuity.com) version 48207413 (Qiagen, Redwood City, CA, USA). Hierarchical cluster analysis of the samples was performed using ArrayTrack HCA and PCA package downloaded from (https://www.fda.gov/science-research/bioinformatics-tools/arraytracktm-hca-pca-standalone-package-powerful-data-exploring-tools) (FDA, Jefferson, AZ, USA) utilizing the Euclidean distance and the Ward‘s linkage type of the expression matrix. Heatmap was generated using the HeatmapViewer module in GenePattern [17]. Gene list comparison were done using Venny 2.1.0 [18].

### 2.5. Cell Types Enrichment Analysis

Cell types enrichment analysis was performed on the normalized dataset of 20,410 genes using the xCell webtool [19,20], including controls, DLE and SCLE samples.

### 2.6. Statistical Analyses

Gene expression changes in CLE samples were compared to 13 similarly processed healthy control biopsies using the Significance Analysis of Microarrays (SAM) method implemented in the TIGR MultiExperiment Viewer application version 4.9.0 (unpaired analysis) [21]. Genes regulated with a *q*-value (false discovery rate) <0.05 were considered significant and used for further transcriptional and pathway analyses.

## 3. Results

### 3.1. Comparison of DLE vs. SCLE Skin Lesions

**Figure 1:**
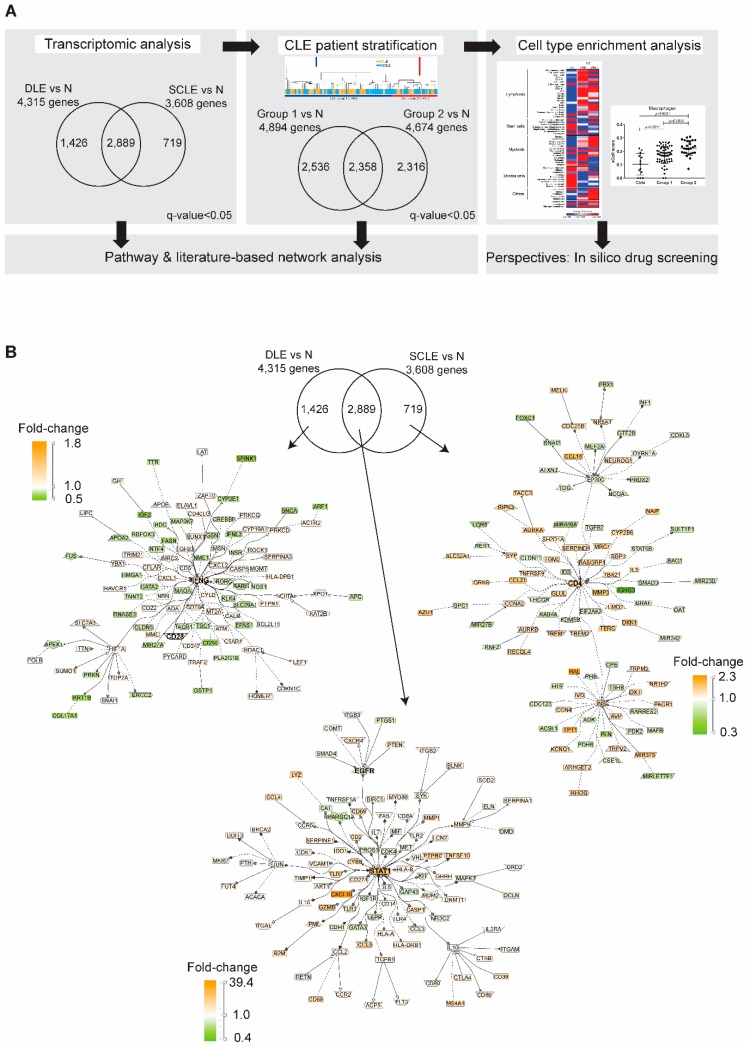
Transcriptomic comparison of DLE and SCLE lesions. (**A**). Graphical representation of the strategy used to analyze CLE lesions at a transcriptional level. (**B**). Literature-based networks (GePS) obtained from the genes regulated in DLE and SCLE vs. normal biopsies. The pictures display the 100 best connected genes co-cited in PubMed abstracts in the same sentence linked to a function word (most relevant genes/interactions). Orange represents the genes that are upregulated and green represents the genes that are downregulated in skin lesions compared to controls.

Despite modifications to the RNA by FFPE treatment, gene expression patterns from formalin fixed, paraffin-embedded (FFPE) tissues provided comparable findings to freshly isolated RNA [22,23,24] (Figure S1). In order to develop a large database of expression changes in both DLE and SCLE, we utilized FFPE biopsies to isolate RNA and to conduct expression profiling using Affymetrix ST2.1 microarrays. 47 DLE and 43 SCLE patients were compared to 13 control skin samples. Clinical data for these patients are available in Table 1. No correlations between DEGs in CLE vs. control samples and medication use were noted except for *PLEK* (r = −0.4511) and *HNRNPH3* (r = −0.4737) with antimalarial use. Analysis strategy is shown in Figure 1A. In order to capture the broadest summary of differentially expressed genes (DEGs) in DLE vs. normal or SCLE vs. normal, we evaluated expression changes using a *q*-value <0.05 and no fold change filter was applied. Literature-based network analysis of the 2889 DEGs shared between DLE and SCLE identified upregulation of *STAT1* (fold-change = 8 in DLE and 6 in SCLE) and down-regulation of *EGFR* (fold- change = 0.6 in both lesion type) genes as primary nodes. For DEGs unique to DLE, nodes were identified involving interferon (IFN)-γ signaling and T cell co-stimulation (CD28) (Figure 1B-left panel). Fewer numbers of DEGs were identified that were unique to SCLE itself but upregulation of CD4 was noted in SCLE lesions (Figure 1B-right panel). Unique and shared canonical pathways were denoted using Ingenuity Pathway Analysis software in Table 2. Numerous transcription factors were regulated in each of the skin biopsy groups. Genomatix Pathway Systems software was used to determine whether these transcription factors were potentially regulating DEGs in each subtype. As shown in Table 3, amongst the DEG shared by both DLE and SCLE lesions, STAT1 and IRF1 binding sites were predicted to be present in the promoters of 226 and 157 (respectively) DEGs (*q*-value <0.05, absolute log2 fold-change ≥1.5). Other important transcription factors for DEG common to DLE and SCLE included SPI1, IRF7, IRF8, STAT2, and IRF9. A specific listing of which shared DEGs are regulated by STAT1 and IRF1 is shown in Supplemental Table S1.

### 3.2. Characterization of Lesions of CLE-Only vs. SLE Patients

**Figure 2:**
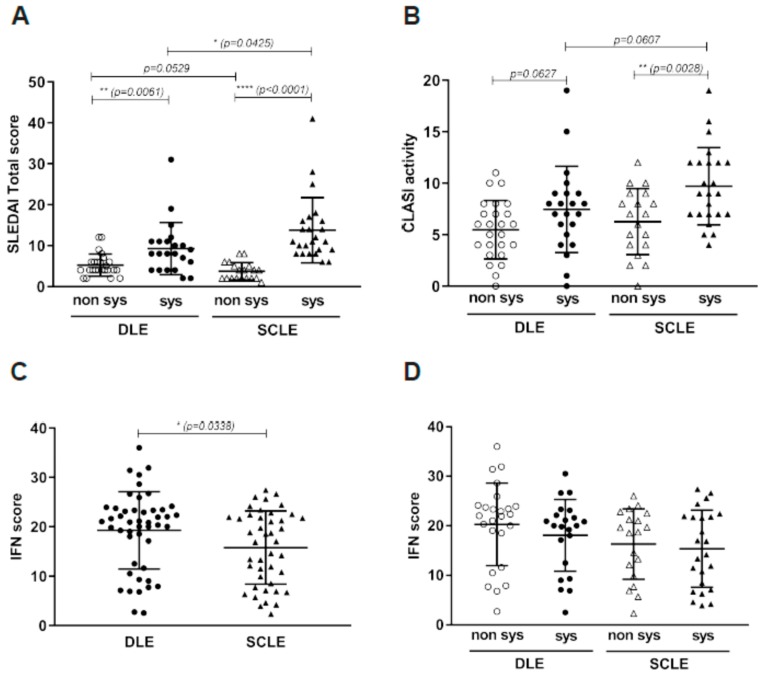
Disease activity in CLE lesions. (**A**). SLEDAI total score is higher is systemic compared to non-systemic disease in both DLE and SCLE lesions. (**B**). CLASI activity is higher in systemic compared to non-systemic disease in both DLE and SCLE. (**C**). IFN score was modestly but significantly lower in SCLE compared to DLE lesions. (**D**). IFN score did not show to be significantly different in systemic and non-systemic disease in patients showing DLE and SCLE lesions.

CLE can be found as a disease entity itself or as a manifestation of SLE. As denoted in Figure 2A, systemic disease activity, as measured by SLEDAI, was higher in patients with SLE-associated disease, as expected. Cutaneous lupus erythematosus disease area and severity index (CLASI [12]) scores were significantly higher in SCLE patients with systemic disease as well. DLE patients with SLE had a non-significant trend towards increased CLASI activity (Figure 2B). Type I IFNs have been reported as an important signaling pathway in CLE pathogenesis [4,18] and circulating IFN scores may correlate with CLASI [25]. Our data also suggest that IFNs are a central component to both DLE and SCLE lesions (Figure 1); thus, we compared IFN scores [20] of six IFN-regulated genes in CLE skin between lesion types. Overall, both subtypes showed elevated IFN scores, but DLE lesions had modest but significantly higher IFN scores than SCLE lesions (Figure 2C); no differences were seen when the data was divided amongst systemic vs. non-systemic disease-associated lesions (Figure 2D).

We then compared DEG between DLE with (*n* = 22) and without (*n* = 25) systemic disease and only nine downregulated genes were found between the groups. A similar lack of difference was seen when SCLE lesions from CLE-only (*n* = 19) vs. SLE patients (*n* = 24) were compared (DEG = 0). These data suggest that the pathophysiology of DLE and SCLE lesions is similar whether they occur in patients with or without systemic disease.

### 3.3. Molecular Subtyping of CLE Lesions

**Figure 3:**
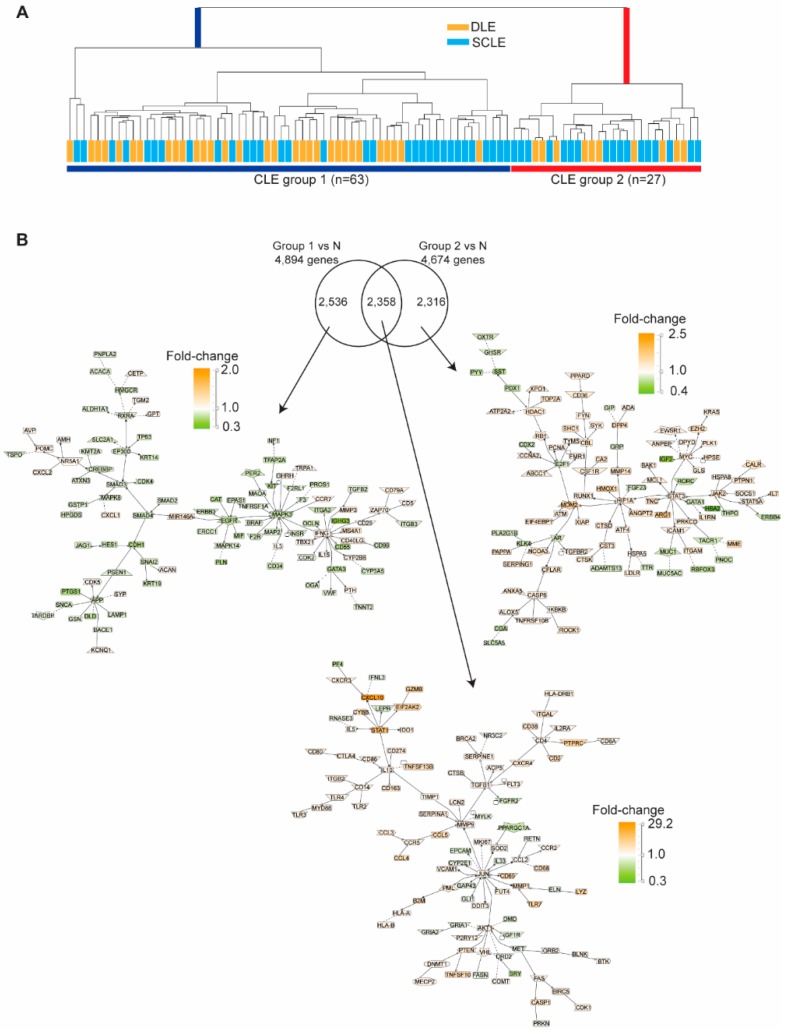
Molecular subtyping of CLE lesions. (**A**). Hierarchical clustering of all CLE lesions showed two distinct subgroups, not classified by lesion type, nor disease activity. (**B**). Literature-based networks from the CLE subgroup comparison.

**Figure 4:**
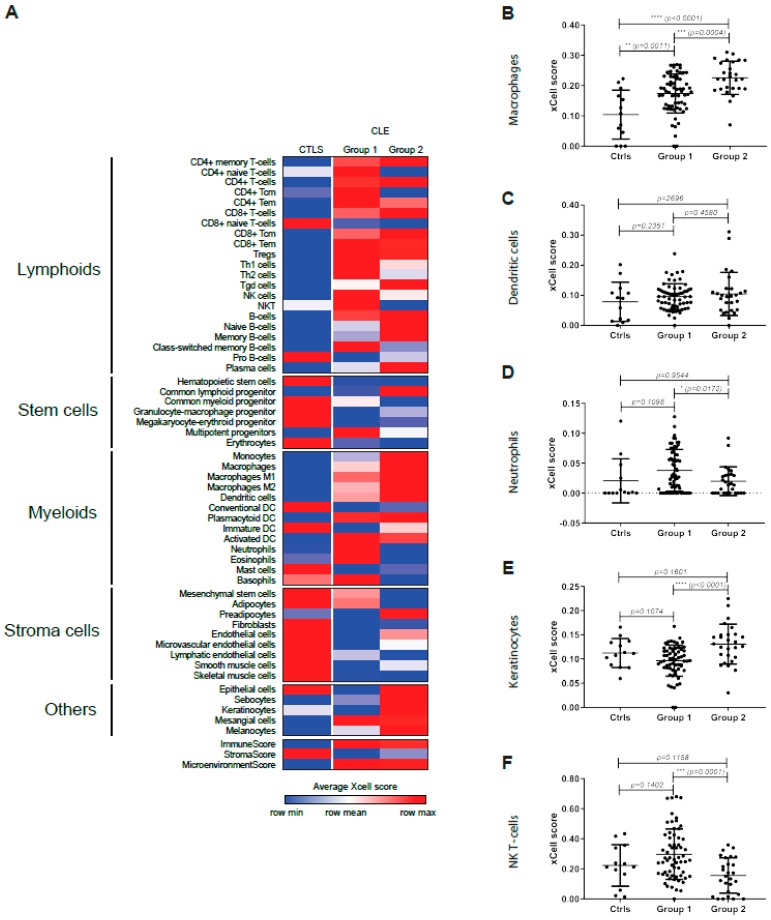
Cell type enrichment analysis using xCell tool. (**A**). Heatmap of relevant cell types representing average xCell score for each CLE subgroups compared to controls. (**B**). Graph of the macrophage xCell scores, showing a significantly higher score in CLE Group 2 compared to Group 1 and controls. (**C**). Graph of the dendritic cell xCell scores. (**D**). Graph of the neutrophil xCell scores, showing a significant lower score in CLE Group 2 compared to Group 1. (**E**). Graph of the keratinocyte xCell scores, showing a significant higher score in CLE group 2 compared to Group 1. (**F**). Graph of the NKT xCell scores, showing a significant higher score in CLE Group 1 compared to Group 2. For (B–F), comparisons were made via unpaired Students’ *t*-test.

Given that the majority of DEG genes were shared between DLE and SCLE lesions and that no differences were seen in CLE-only vs. SLE-associated lesions, we then opted to utilize hierarchical clustering to evaluate whether CLE lesions could be subtyped through their molecular signatures alone (Figure 3A). Two main clusters were identified that were significantly separated by IFN score and body mass index (BMI) but not by systemic or cutaneous disease activity (Table 4). Strikingly, DLE and SCLE patients were equally distributed between the two subgroups (Table 4). Literature- based network analysis of the DEG between each subgroup vs. controls (*q*-value <0.05) revealed *STAT1*, *IL10*, *MMP9*, *TGFB1,* and *JUN* as shared major nodes and the activation of immune cell canonical pathways (Figure 3B, middle panel). The gene expression networks altered only in CLE Group 1 included the up-regulation of *IFNG* and the down-regulation of *EGFR* and *MAPK3* (Figure 3B, left panel). *JAK2*, *STAT3,* and *HIF1A* networks were up-regulated only in CLE Group 2 (Figure 3B, right panel). To characterize the potential cell types involved in the two CLE subgroups, we utilized xCell, a program which generates cell type enrichment scores using bulk gene expression data. Intriguingly, Group 1 was characterized by a significantly higher granulocytic signature and increased natural killer (NK) and NKT cells whereas Group 2 demonstrated increased expression of myeloid (monocytes and macrophages) and keratinocyte-expressed genes (Figure 4A–F). These data suggest that signaling and neutrophilic vs. monocyte/epidermal phenotypes may be distinguishing features of CLE lesions that may be useful to guide treatment protocols.

## 4. Discussion

CLE is a relatively rare but disfiguring condition that significantly affects quality of life [26]. Discoid and SCLE lesions have different clinical presentations, yet both can be refractory to therapy and result in lower quality of life for patients [27,28]. Understanding the pathobiology of CLE is thus important to inform treatment options. This study defines the gene expression differences between the two most common subtypes of CLE: DLE and SCLE. Importantly, the majority of DEG were noted to be in common between these subtypes and only a node for IFNγ was a unique feature in DLE lesions. Remarkably, unsupervised clustering led to division of CLE lesions into two subgroups defined by IFNγ vs. STAT3/JAK2 signaling. Cell type enrichment analysis of the data identified the clusters to be primarily defined by granulocytic (neutrophil and eosinophil) signatures vs. monocytic and epidermal signatures. These data suggest that further studies should consider molecular signatures of the skin itself when considering therapy development.

Our data confirm the notion that type I IFN regulated pathways are increased in both DLE and SCLE patients [25]. Others have noted a correlation with IFN signatures and disease activity in SCLE [25]. Here, we did not see a correlation with IFN signature and CLASI or SLEDAI, but rather the cutaneous IFN signature was elevated in every skin sample compared to control. Previous work has identified elevated IFN-κ and IFN-α as the primary IFNs present in the lesions of these patients [4] and IFN-κ as a driver of skewed inflammatory responses in SLE keratinocytes [20]. Further work into the role of IFNs in SLE skin is ongoing by several groups.

Another common theme noted in CLE lesions is repression of the EGFR signaling pathways. This data fits with work in non-lesional SLE skin which has identified absence of Langerhans cells in SLE skin. Langerhans cells are required for derivation of EGFR signals to promote keratinocyte survival [29]. Further work to understand what regulates Langerhans cell numbers in SLE skin will be important for understanding CLE pathogenesis.

Neutrophils are an important part of systemic lupus pathogenesis [30,31] and our data suggests that at least some patients also display an elevated neutrophilic signature in their skin. Other bioinformatic work has also identified enrichment of neutrophil signatures in whole blood of pediatric SLE patients with associated skin disease [30]. Murine studies have not identified a causative role for neutrophils in IgG induced skin lesions, but the death of neutrophils by apoptosis [32] or NETosis in the skin [33] may be important contributors to disease. Longitudinal trials are required to identify whether this signature may correlate with treatment response to neutrophil- targeting medications such as dapsone or whether the neutrophilic signature is more reflective of the age of lesions or a photosensitive response [34]. The other cluster of CLE patients expressed genes identified with monocytic-derived cell populations such as DCs and also an increased keratinocyte signature that may relate to hyperkeratosis or increased epidermal turnover [35]. At this time, it is unknown whether the keratinocyte differences are able to result in recruitment of myeloid-derived cells to the skin. Certainly, SLE keratinocytes are able to activate dendritic cells in an IFN dependent manner [4], but again, longitudinal studies to evaluate the evolution of this signature and how it changes over time and with treatment are required for further insight. Intriguingly, the Group 2 population also demonstrated upregulation of STAT3, STAT5, and JAK2 signaling, which suggests that drugs which target JAK/STAT pathways would have potential efficacy in this subgroup of patients.

## 5. Conclusions

In conclusion, our work suggests that while we can find differences in CLE lesional subtypes, particularly an IFN-γ signature in DLE patients, molecular signatures that center around neutrophilic or monocytic subtypes may be more informative regarding pathobiology and relevant targets for treatment. Ongoing work in this arena will provide longitudinal data for further confirmation of these studies and provide better therapeutic options for this devastating disorder.

## Figures and Tables

**Table 1:** Summary Characteristics of lupus patients included in the microarray study. Comparisons between DLE and SCLE were made using two-sided unpaired Students’ *t*-test.

	DLE (*n* = 47)	SCLE (*n* = 43)	*p* Value
Mean Age (±SEM)	45.2 (2.0)	47.2 (2.7)	0.5590
Gender (% Female)	78.7	83.7	0.5504
Ethnicity (% African-American)	40.4	4.7	<0.0001
BMI (±SEM)	28.0 (0.9)	27.6 (1.2)	0.8022
SLEDAI (±SEM)	7.1 (0.8)	9.3 (1.2)	0.1180
CLASI Activity (±SEM)	6.4 (0.5)	8.2 (0.6)	0.0272
CLASI Damage (±SEM)	4.2 (0.7)	0.7 (0.3)	0.0001
% ≥4 ACR criteria for SLE within 3 years of biopsy	46.8	55.8	0.1541
Positive anti-Smith antibodies (%)	23.4	25.6	0.8981
Positive anti-Ro antibodies (%)	31.9	58.1	0.0308
Positive antiphospholipid antibodies (%)	10.6	25.6	0.1342
dsDNA titer (±SEM)	9.6 (2.8)	35.0 (13.4)	0.0703
IFN score (±SEM)	19.3 (0.2)	15.8 (0.2)	0.0338
Medications (% on drug at time of biopsy)			
Oral Prednisone <10 mg daily	8.5	4.7	0.4690
Oral Prednisone >10 mg daily	10.6	23.3	0.1111
Mycophenolate Mofetil	2.1	11.6	0.0725
Antimalarials	40.4	44.2	0.7219
Methotrexate	2.1	0	0.3417

**Table 2:** Ingenuity Pathway Analysis: Top 10 canonical pathways from the genes uniquely regulated in DLE, SCLE and in both lesion types compared to controls (the total number of genes in each pathway is indicated in brackets).

**From the 1426 Genes Uniquely Regulated in DLE Compared to Controls**		***p*-Value**	**Number of Regulated Genes**
1	Phospholipase C Signaling (238)	7.6 × 10^−5^	29
2	Epithelial Adherens Junction Signaling (149)	9.3 × 10^−5^	21
3	×enobiotic Metabolism Signaling (276)	9.3 × 10^−4^	29
4	Regulation of IL-2 E×pression in Activated and Anergic T Lymphocytes (86)	1.0 × 10^−3^	13
5	Glucocorticoid Receptor Signaling (344)	1.0 × 10^−3^	34
6	NF-κB Signaling (182)	1.4 × 10^−3^	21
7	Systemic Lupus Erythematosus Signaling (209)	1.6 × 10^−3^	23
8	PKCθ Signaling in T Lymphocytes (161)	1.8 × 10^−3^	19
9	Granzyme A Signaling (17)	1.9 × 10^−3^	5
10	T Cell Receptor Signaling (117)	2.3 × 10^−3^	15
**From the 2889 Genes Shared in DLE and SCLE Compared to Controls**		***p*-Value**	**Number of Regulated Genes**
1	Communication between Innate and Adaptive Immune Cells (80)	5.0 × 10^−13^	34
2	Th1 and Th2 Activation Pathway (175)	3.2 × 10^−11^	51
3	Crosstalk between Dendritic Cells and Natural Killer Cells (86)	1.7 × 10^−10^	32
4	Pathogenesis of Multiple Sclerosis (9)	2.3 × 10^−9^	9
5	Dendritic Cell Maturation (179)	2.8 × 10^−9^	48
6	Role of Pattern Recognition Receptors in Recognition of Bacteria and Viruses (158)	3.6 × 10^−9^	44
7	Granulocyte Adhesion and Diapedesis (160)	5.5 × 10^−9^	44
8	Th2 Pathway (141)	1.0 × 10^−8^	40
9	Th1 Pathway (125)	1.0 × 10^−8^	37
10	T Cell E×haustion Signaling Pathway (176)	4.2 × 10^−8^	45
**From the 719 Genes Uniquely Regulated in SCLE Compared to Controls**		***p*-Value**	**Number of Regulated Genes**
1	Role of Oct4 in Mammalian Embryonic Stem Cell Pluripotency (44)	1.2 × 10^−3^	6
2	Cancer Drug Resistance by Drug Efflu× (58)	5.1 × 10^−3^	6
3	Hematopoiesis from Pluripotent Stem Cells (37)	1.8 × 10^−2^	4
4	Dermatan Sulfate Biosynthesis (Late Stages) (41)	2.6 × 10^−2^	4
5	Glutamine Biosynthesis I (1)	2.8 × 10^−2^	1
6	Adenine and Adenosine Salvage VI (1)	2.8 × 10^−2^	1
7	Protein Ubiquitination Pathway (263)	3.1 × 10^−2^	13
8	Antiproliferative Role of TOB in T Cell Signaling (26)	3.4 × 10^−2^	3
9	Dolichyl-diphosphooligosaccharide Biosynthesis (11)	3.5 × 10^−2^	2
10	Protein Kinase A Signaling (383)	3.7 × 10^−2^	17

**Table 3:** Transcription factor (TF) analysis as assessed by GePS.

Transcription Factor	ENTREZ GENE ID	Number of Genes Having a Potential Binding Site in Their Promoter for the Indicated Transcription Factor in the 1335 Genes Regulated in Both DLE and SCLE vs Normal Biopsies (*q*-value <0.05, Absolute Log Fold-Change ≥0.6)	DLE Fold-Change	DLE *q*-Value	SCLE Fold-Change	SCLE *q*-Value
STAT1	6772	226	8.1	0.0000	6.1	0.0000
IRF1	3659	157	2.7	0.0000	2.2	0.0000
GATA3	2625	132	0.5	0.0012	0.7	0.0254
IRF7	3665	122	1.6	0.0000	1.6	0.0010
SPI1	6688	102	1.9	0.0000	2.0	0.0000
IRF8	3394	93	2.3	0.0000	2.1	0.0000
STAT4	6775	88	1.9	0.0000	1.5	0.0021
STAT2	6773	74	3.1	0.0000	2.4	0.0000
IRF9	10379	64	1.7	0.0000	1.8	0.0000
IKZF1	10320	56	1.9	0.0000	1.9	0.0000
PML	5371	55	1.8	0.0000	1.8	0.0000
SRY	6736	33	0.3	0.0020	0.3	0.0009
EBF1	1879	30	0.6	0.0077	0.6	0.0073
PLAGL1	5325	26	0.5	0.0024	0.6	0.0051
SOX5	6660	25	0.4	0.0000	0.4	0.0000
IKZF3	22806	25	2.2	0.0000	1.8	0.0000
E2F3	1871	25	1.6	0.0000	1.7	0.0000
IRF6	3664	23	0.6	0.0000	0.7	0.0056
NFIA	4774	23	0.5	0.0000	0.5	0.0000
THRA	7067	22	0.5	0.0000	0.6	0.0016
NFIB	4781	19	0.6	0.0036	0.6	0.0159
TBX5	6910	18	0.6	0.0020	0.7	0.0159
NR1D1	9572	18	0.4	0.0000	0.4	0.0000
HSF2	3298	17	0.6	0.0000	0.6	0.0000
EMX2	2018	14	0.5	0.0000	0.5	0.0000
SIX3	6496	13	0.6	0.0004	0.6	0.0011
TFAP2B	7021	12	0.6	0.0007	0.6	0.0009
TCF7L1	83439	11	0.6	0.0027	0.7	0.0116
PEG3	5178	10	0.6	0.0008	0.6	0.0000
FOXN3	1112	10	0.6	0.0007	0.7	0.0103
PAX9	5083	9	0.6	0.0045	0.7	0.0228
GRHL2	79977	6	0.6	0.0007	0.6	0.0029
TFEC	22797	6	2.5	0.0000	2.1	0.0000
HOXC10	3226	5	0.4	0.0000	0.6	0.0066
NFIX	4784	5	0.5	0.0000	0.5	0.0021
PPARGC1A	10891	5	0.4	0.0000	0.5	0.0009
HOXC4	3221	3	0.5	0.0000	0.7	0.0121
MESP1	55897	3	0.6	0.0014	0.6	0.0111
POU2F3	25833	3	0.4	0.0000	0.5	0.0021
ZNF704	619279	2	0.5	0.0000	0.6	0.0000
HLF	3131	1	0.5	0.0000	0.6	0.0131

**Table 4:** Summary characteristics of lupus patients in each defined subgroup. Comparisons between the two subgroups were made using two-sided unpaired Students’ *t*-test.

	Group 1 (*n* = 63)	Group 2 (*n* = 27)	*p* Value
Mean Age (±SEM)	46.4 (1.9)	45.6 (3.4)	0.8286
Gender (% Female)	79.4	85.2	0.5234
Ethnicity (% African-American)	23.8	22.2	0.8722
BMI (±SEM)	28.9 (0.9)	25.1 (0.8)	0.0161
% of DLE—% of SCLE	49.2–50.8	59.3–40.7	0.3873
SLEDAI (±SEM)	8.7 (0.9)	6.9 (1.0)	0.3221
CLASI Activity (±SEM)	7.3 (0.4)	7.1 (0.9)	0.9102
CLASI Damage (±SEM)	2.0 (0.4)	3.8 (1.0)	0.0408
% ≥4 ACR criteria for SLE within 3 years of biopsy	55.6	40.7	0.2531
Positive anti-Smith antibodies (%)	20.6	33.3	0.1280
Positive anti-Ro antibodies (%)	41.3	51.9	0.2640
Positive antiphospholipid antibodies (%)	23.8	3.7	0.0705
dsDNA titer (±SEM)	22.2 (8.5)	21.6 (10.8)	0.9718
IFN score (±SEM)	16.4 (0.9)	20.3 (1.6)	0.0289
Medications (%)			
Oral Prednisone <10 mg daily	7.9	3.7	0.4663
Oral Prednisone >10 mg daily	14.3	22.2	0.3602
Mycophenolate Mofetil	9.5	0.0	0.0991
Antimalarials	47.6	29.6	0.1159
Methotrexate	1.6	0.0	0.5158

## Supplementary Materials

The following are available online at https://www.mdpi.com/2077-0383/8/8/1244/s1. Figure S1: There is a strong correlation between expression profiles of RNA isolated from FFPE or fresh tissue. Table S1: 226 genes regulated in DLE and SCLE (*q*-value < 0.05, absolute log2 fold-change ≥ 0.6) having a potential binding site for STAT1 in their promoter.

## Abbreviations

BMI	body mass index
cDNA	complementary DNA
CLE	cutaneous lupus erythematosus
DEG	differentially expressed gene
DLE	discoid lupus erythematosus
FFPE	formalin-fixed, paraffin-embedded
IFN	interferon;
SCLE	subacute cutaneous lupus erythematosus
SLE	systemic lupus erythematosus
UV	ultraviolet
