# Supplementary material for: Molecular Profiling of Cutaneous Lupus Lesions Identifies Subgroups Distinct from Clinical Phenotypes
Source: J Clin Med. 2019 Aug 17;8(8):1244. doi: 10.3390/jcm8081244 (PMC6723404; doi:10.3390/jcm8081244)
Supplement: Supplementary file 1 [file jcm-08-01244-s001.pdf]

SUPPLEMENTAL FILES

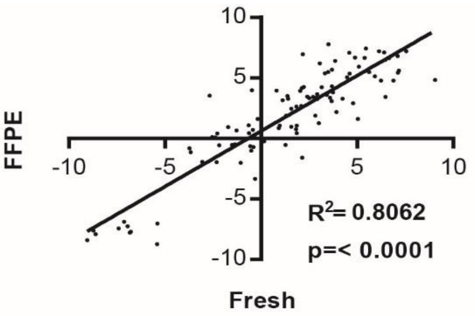

Figure S1

Figure S1: There is a strong correlation between expression profiles of RNA isolated from FFPE or fresh tissue. Murine skin (n=5) was sampled after euthanasia. One half of the skin sample was subjected to fresh RNA isolation and the other was fixed in formalin for 16 hours followed by paraffin embedding prior to RNA isolation. Real-time PCR was used to quantitate RNA levels of 10 different genes and the ddct for each gene was plotted for each RNA isolation subtype. Correlation between FFPE and freshly isolated RNA delta-CTs was done via linear regression using GraphPad Prism v.6.

Supplemental Table S1.

226 genes regulated in DLE and SCLE (q-value<0.05, absolute log2 fold-change ≥ 0.6) having a potential binding site for STAT1 in their promoter					
**Gene symbol**	**ENTREZ GENE ID**	**DLE fold-change**	**DLE q-value**	**SCLE fold-change**	**SCLE q-value**
**FGFR2**	**2263**	0.5	0.0000	0.6	0.0021
**CXCL10**	**3627**	39.5	0.0000	22.5	0.0000
**CCL5**	**6352**	3.6	0.0000	2.7	0.0000
**KRT16**	**3868**	6.4	0.0000	6.1	0.0000
**RTP4**	**64108**	1.9	0.0000	1.6	0.0010
**PLEK**	**5341**	2.5	0.0000	2.0	0.0000
**S100A8**	**6279**	4.5	0.0000	3.4	0.0000
**CD9**	**928**	0.6	0.0016	0.6	0.0109
**IL2RG**	**3561**	2.9	0.0000	2.3	0.0000
**CAT**	**847**	0.6	0.0051	0.6	0.0021
**CD3D**	**915**	2.9	0.0000	2.3	0.0000
**HLA-DRA**	**3122**	2.3	0.0000	1.9	0.0000
**ITGB5**	**3693**	0.7	0.0258	0.6	0.0111
**SERPINB3**	**6317**	4.7	0.0000	4.3	0.0000
**CD274**	**29126**	1.7	0.0051	1.7	0.0121
**IL12RB2**	**3595**	1.7	0.0073	1.6	0.0009
**STK17B**	**9262**	1.9	0.0007	1.6	0.0121
**ITGAX**	**3687**	2.0	0.0000	1.9	0.0000
**ATP5PO**	**539**	0.5	0.0140	0.4	0.0016
**SERPINB4**	**6318**	6.5	0.0000	3.1	0.0000
**HAVCR2**	**84868**	1.9	0.0000	1.9	0.0000

**LILRB1**	**10859**	4.3	0.0000	2.1	0.0021
**ITGB2**	**3689**	1.7	0.0000	1.6	0.0021
**GBP4**	**115361**	4.3	0.0000	2.5	0.0000
**SORBS1**	**10580**	0.5	0.0012	0.6	0.0128
**MYLK**	**4638**	0.6	0.0099	0.6	0.0079
**APOBEC3G**	**60489**	2.6	0.0000	2.5	0.0000
**LY96**	**23643**	2.1	0.0000	1.7	0.0000
**CCL7**	**6354**	3.8	0.0000	3.7	0.0015
**TLR7**	**51284**	4.4	0.0000	3.1	0.0000
**FSCN1**	**6624**	1.8	0.0000	1.8	0.0000
**DEPTOR**	**64798**	0.6	0.0057	0.6	0.0062
**KCNA3**	**3738**	1.6	0.0000	1.6	0.0015
**HLA-C**	**3107**	1.9	0.0000	2.0	0.0000
**PTEN**	**5728**	2.2	0.0000	1.9	0.0016
**HSF2**	**3298**	0.6	0.0000	0.6	0.0000
**FGL2**	**10875**	2.1	0.0000	1.8	0.0026
**AJUBA**	**84962**	0.6	0.0042	0.6	0.0103
**CD80**	**941**	1.7	0.0016	1.7	0.0010
**CD163**	**9332**	1.9	0.0000	2.4	0.0000
**KLRC1**	**3821**	1.7	0.0061	1.6	0.0037
**IVL**	**3713**	2.2	0.0166	2.0	0.0220
**CXCL13**	**10563**	2.8	0.0000	1.8	0.0009
**WAS**	**7454**	1.5	0.0012	1.8	0.0000
**SOX5**	**6660**	0.4	0.0000	0.4	0.0000
**CH25H**	**9023**	1.8	0.0000	1.5	0.0051
**AKT1**	**207**	1.6	0.0145	1.7	0.0010
**CRLF2**	**64109**	0.5	0.0000	0.5	0.0037
**UBD**	**10537**	2.2	0.0000	1.9	0.0011
**SAMHD1**	**25939**	2.6	0.0000	2.2	0.0000
**CCL8**	**6355**	2.3	0.0000	1.8	0.0063
**CYBB**	**1536**	3.3	0.0000	2.4	0.0000
**CLEC4A**	**50856**	1.6	0.0117	1.5	0.0410
**RPS5**	**6193**	0.4	0.0000	0.4	0.0000
**ACP1**	**52**	0.6	0.0007	0.6	0.0015
**CADM1**	**23705**	0.6	0.0068	0.6	0.0199
**RGS1**	**5996**	4.3	0.0000	2.0	0.0000
**IFI44**	**10561**	20.5	0.0000	13.7	0.0000
**VCAM1**	**7412**	1.8	0.0000	1.6	0.0021
**GBP1**	**2633**	7.8	0.0000	5.4	0.0000
**SELL**	**6402**	1.8	0.0013	1.5	0.0019
**ICOS**	**29851**	1.5	0.0129	1.5	0.0055
**CCL4**	**6351**	2.9	0.0000	2.4	0.0000

**BLNK**	**29760**	1.8	0.0000	1.6	0.0073
**S100A9**	**6280**	8.6	0.0000	7.8	0.0000
**RAC2**	**5880**	2.8	0.0000	2.2	0.0000
**MMP1**	**4312**	2.3	0.0233	1.8	0.0327
**TYROBP**	**7305**	2.5	0.0000	2.4	0.0000
**GZMB**	**3002**	4.4	0.0000	3.7	0.0000
**POSTN**	**10631**	0.5	0.0077	0.4	0.0022
**CRTAM**	**56253**	1.7	0.0008	1.5	0.0050
**CTSL**	**1514**	1.7	0.0117	1.9	0.0374
**SRY**	**6736**	0.3	0.0020	0.3	0.0009
**EGFR**	**1956**	0.6	0.0037	0.6	0.0254
**RNASE2**	**6036**	0.6	0.0117	0.6	0.0067
**GPR183**	**1880**	3.5	0.0000	2.5	0.0000
**CD48**	**962**	2.8	0.0000	2.3	0.0000
**FPR1**	**2357**	1.6	0.0007	1.7	0.0247
**CD2**	**914**	3.2	0.0000	2.4	0.0000
**SPI1**	**6688**	1.9	0.0000	2.0	0.0000
**PTPN22**	**26191**	2.4	0.0000	2.0	0.0000
**GBP2**	**2634**	1.9	0.0000	1.8	0.0011
**TNFSF13B**	**10673**	3.1	0.0000	2.1	0.0000
**GZMA**	**3001**	3.6	0.0000	2.4	0.0000
**PTPN6**	**5777**	1.8	0.0000	1.6	0.0000
**PTPRC**	**5788**	4.8	0.0000	3.2	0.0000
**IKZF3**	**22806**	2.2	0.0000	1.8	0.0000
**LGMN**	**5641**	1.7	0.0000	1.6	0.0121
**E2F3**	**1871**	1.6	0.0000	1.7	0.0000
**KIT**	**3815**	0.5	0.0017	0.6	0.0159
**GAP43**	**2596**	0.6	0.0000	0.6	0.0016
**SLC25A6**	**293**	0.6	0.0007	0.6	0.0026
**CD69**	**969**	3.4	0.0000	2.1	0.0000
**C1QB**	**713**	2.5	0.0000	2.3	0.0000
**ITGAL**	**3683**	1.9	0.0000	1.8	0.0011
**PPARGC1A**	**10891**	0.4	0.0000	0.5	0.0009
**FCGRT**	**2217**	0.6	0.0000	0.6	0.0016
**CD38**	**952**	1.9	0.0000	1.7	0.0019
**SIGLEC1**	**6614**	1.5	0.0036	1.8	0.0000
**TLR3**	**7098**	1.9	0.0000	1.5	0.0176
**VAV1**	**7409**	1.5	0.0000	1.6	0.0009
**FTH1**	**2495**	1.8	0.0007	1.6	0.0394
**PYHIN1**	**149628**	2.7	0.0000	1.9	0.0000
**PIK3CG**	**5294**	1.9	0.0000	1.5	0.0017
**LYZ**	**4069**	4.3	0.0000	3.7	0.0000

**TCF7L1**	**83439**	0.6	0.0027	0.7	0.0116
**PVT1**	**5820**	1.6	0.0159	1.5	0.0426
**TNFRSF17**	**608**	1.9	0.0000	1.6	0.0148
**RPS6**	**6194**	0.6	0.0073	0.6	0.0116
**CCL3**	**6348**	1.7	0.0068	1.6	0.0176
**CCL19**	**6363**	2.1	0.0056	1.7	0.0185
**MNDA**	**4332**	2.5	0.0000	2.2	0.0000
**CXCL11**	**6373**	12.7	0.0000	6.8	0.0000
**CLDN1**	**9076**	0.5	0.0007	0.6	0.0062
**VHL**	**7428**	1.5	0.0021	1.5	0.0085
**EPCAM**	**4072**	0.7	0.0030	0.6	0.0010
**CTLA4**	**1493**	1.7	0.0000	1.8	0.0011
**CCL27**	**10850**	0.4	0.0016	0.6	0.0311
**GATA3**	**2625**	0.5	0.0012	0.7	0.0254
**IKZF1**	**10320**	1.9	0.0000	1.9	0.0000
**FCGR1B**	**2210**	0.1	0.0000	0.2	0.0000
**CASP1**	**834**	2.3	0.0000	2.1	0.0000
**SCGB3A1**	**92304**	0.4	0.0012	0.4	0.0011
**SAMD9L**	**219285**	5.8	0.0000	3.7	0.0000
**TIMP3**	**7078**	0.5	0.0000	0.6	0.0043
**HIST1H2BJ**	**8970**	1.7	0.0051	2.2	0.0000
**CD1A**	**909**	0.5	0.0000	0.4	0.0000
**CLEC7A**	**64581**	2.6	0.0000	2.6	0.0000
**IL7R**	**3575**	8.7	0.0000	4.7	0.0000
**ISG15**	**9636**	8.6	0.0000	7.5	0.0000
**PIAS3**	**10401**	0.6	0.0016	0.6	0.0000
**PDCD1LG2**	**80380**	1.8	0.0000	1.7	0.0000
**PHLPP1**	**23239**	0.6	0.0000	0.7	0.0051
**EBF1**	**1879**	0.6	0.0077	0.6	0.0073
**CD68**	**968**	2.8	0.0000	2.5	0.0000
**BST2**	**684**	7.6	0.0000	4.6	0.0000
**NMI**	**9111**	3.3	0.0000	2.6	0.0000
**ITK**	**3702**	1.7	0.0012	1.7	0.0019
**IFIT1**	**3434**	7.0	0.0000	4.7	0.0000
**UBE2L6**	**9246**	2.7	0.0000	2.3	0.0000
**GBP5**	**115362**	7.5	0.0000	3.5	0.0000
**EIF2AK2**	**5610**	4.5	0.0000	2.9	0.0000
**TNFSF10**	**8743**	3.2	0.0000	2.3	0.0000
**B2M**	**567**	2.7	0.0000	2.5	0.0000
**PARP9**	**83666**	3.8	0.0000	3.1	0.0000
**IFIH1**	**64135**	6.5	0.0000	4.4	0.0000
**ISG20**	**3669**	1.8	0.0008	1.5	0.0058

**SCO2**	**9997**	1.9	0.0000	2.0	0.0000
**IRF1**	**3659**	2.7	0.0000	2.2	0.0000
**STAT2**	**6773**	3.1	0.0000	2.4	0.0000
**MLKL**	**197259**	2.2	0.0000	1.9	0.0000
**LCN2**	**3934**	1.7	0.0000	1.6	0.0070
**PLSCR1**	**5359**	2.3	0.0000	2.0	0.0017
**CDH1**	**999**	0.6	0.0077	0.6	0.0099
**LEPR**	**3953**	0.6	0.0027	0.6	0.0111
**AIM2**	**9447**	3.8	0.0000	2.7	0.0000
**DTX3L**	**151636**	4.8	0.0000	3.5	0.0000
**CD53**	**963**	2.8	0.0000	2.0	0.0000
**IL10RA**	**3587**	1.7	0.0008	1.8	0.0009
**TAP1**	**6890**	1.7	0.0031	1.5	0.0063
**RSAD2**	**91543**	4.2	0.0000	3.2	0.0000
**ADORA1**	**134**	0.4	0.0000	0.6	0.0031
**SECTM1**	**6398**	1.6	0.0180	1.8	0.0010
**OAS1**	**4938**	8.9	0.0000	6.1	0.0000
**DDX60**	**55601**	2.5	0.0000	2.0	0.0009
**IFI44L**	**10964**	23.3	0.0000	15.3	0.0000
**PARP12**	**64761**	2.9	0.0000	2.3	0.0000
**IFIT2**	**3433**	5.0	0.0000	3.4	0.0000
**CD55**	**1604**	0.5	0.0042	0.6	0.0211
**CSK**	**1445**	1.7	0.0000	1.9	0.0000
**IRF8**	**3394**	2.3	0.0000	2.1	0.0000
**SLFN5**	**162394**	2.2	0.0000	1.5	0.0455
**NFIA**	**4774**	0.5	0.0000	0.5	0.0000
**SAMSN1**	**64092**	2.1	0.0000	1.9	0.0000
**CCR1**	**1230**	2.5	0.0000	2.3	0.0000
**STAT4**	**6775**	1.9	0.0000	1.5	0.0021
**CTSS**	**1520**	3.1	0.0000	2.5	0.0000
**IFI6**	**2537**	6.3	0.0000	5.0	0.0000
**MX2**	**4600**	5.0	0.0000	3.3	0.0000
**BIRC3**	**330**	3.8	0.0000	2.6	0.0000
**TRIM22**	**10346**	3.5	0.0000	3.0	0.0000
**EPSTI1**	**94240**	6.6	0.0000	4.3	0.0000
**HERC5**	**51191**	7.5	0.0000	3.9	0.0000
**MAP3K8**	**1326**	1.6	0.0013	1.5	0.0021
**IFI16**	**3428**	3.0	0.0000	2.2	0.0000
**OASL**	**8638**	2.4	0.0000	2.3	0.0000
**DDX5**	**1655**	1.7	0.0000	1.5	0.0016
**WARS**	**7453**	3.0	0.0000	2.8	0.0000
**MDM2**	**4193**	1.8	0.0018	1.6	0.0268

**ADAR**	**103**	1.7	0.0004	1.7	0.0011
**IFIT5**	**24138**	1.9	0.0000	1.8	0.0000
**LYN**	**4067**	2.2	0.0000	1.8	0.0009
**ADAP2**	**55803**	2.0	0.0012	1.8	0.0000
**CD83**	**9308**	1.7	0.0023	1.5	0.0078
**STAT1**	**6772**	8.1	0.0000	6.1	0.0000
**LGALS3BP**	**3959**	1.9	0.0000	1.8	0.0000
**MIF**	**4282**	0.7	0.0356	0.7	0.0354
**CGAS**	**115004**	2.0	0.0000	1.7	0.0000
**MX1**	**4599**	10.3	0.0000	6.9	0.0000
**CFB**	**629**	1.9	0.0000	2.1	0.0000
**IFI30**	**10437**	1.9	0.0000	1.9	0.0000
**PSMB9**	**5698**	1.7	0.0000	1.6	0.0010
**IFI27**	**3429**	3.3	0.0000	3.2	0.0000
**DDX58**	**23586**	5.2	0.0000	3.7	0.0000
**OAS3**	**4940**	7.4	0.0000	5.0	0.0000
**FYB1**	**2533**	3.1	0.0000	2.0	0.0000
**IRF9**	**10379**	1.7	0.0000	1.8	0.0000
**FADS2**	**9415**	0.4	0.0409	0.3	0.0366
**PARP14**	**54625**	7.1	0.0000	5.3	0.0000
**RACK1**	**10399**	0.5	0.0004	0.5	0.0000
**XAF1**	**54739**	4.1	0.0000	3.1	0.0000
**OAS2**	**4939**	5.2	0.0000	4.1	0.0000
**IRF7**	**3665**	1.6	0.0000	1.6	0.0010
**TRIM5**	**85363**	2.0	0.0000	1.6	0.0116
**IFIT3**	**3437**	9.5	0.0000	5.9	0.0000
**CXCL9**	**4283**	22.1	0.0000	11.1	0.0000
**ZC3HAV1**	**56829**	1.9	0.0000	1.6	0.0045
**NLRC5**	**84166**	1.8	0.0000	1.7	0.0000
**DUSP6**	**1848**	1.5	0.0007	1.6	0.0128
**PML**	**5371**	1.8	0.0000	1.8	0.0000
**USP18**	**11274**	4.3	0.0000	3.9	0.0000
**APOL1**	**8542**	4.4	0.0000	3.9	0.0000
**PMP22**	**5376**	0.6	0.0014	0.5	0.0022
**IFITM3**	**10410**	1.6	0.0027	1.7	0.0022
**IFI35**	**3430**	1.7	0.0016	1.6	0.0116
**IFITM1**	**8519**	4.4	0.0000	3.5	0.0000

**157 genes regulated in DLE and SCLE (q-value<0.05, absolute log2 fold-change ≥ 0.6) having a potential binding site for IRF1 in their promoter**					
Gene symbol	ENTREZ GENE ID	DLE fold-change	DLE q-value	SCLE fold-change	SCLE q-value

**CXCL10**	**3627**	39.5	0.0000	22.5	0.0000
**CCL5**	**6352**	3.6	0.0000	2.7	0.0000
**PI3**	**5266**	6.0	0.0000	8.1	0.0000
**PLEK**	**5341**	2.5	0.0000	2.0	0.0000
**S100A8**	**6279**	4.5	0.0000	3.4	0.0000
**ALOX5AP**	**241**	2.1	0.0000	1.6	0.0016
**PDCD1LG2**	**80380**	1.8	0.0000	1.7	0.0000
**CD68**	**968**	2.8	0.0000	2.5	0.0000
**CD3D**	**915**	2.9	0.0000	2.3	0.0000
**SERPINB3**	**6317**	4.7	0.0000	4.3	0.0000
**MYH11**	**4629**	0.3	0.0013	0.4	0.0029
**IL12RB2**	**3595**	1.7	0.0073	1.6	0.0009
**ITGB2**	**3689**	1.7	0.0000	1.6	0.0021
**ACKR3**	**57007**	0.7	0.0024	0.6	0.0016
**CYTH4**	**27128**	1.6	0.0024	1.7	0.0011
**GBP5**	**115362**	7.5	0.0000	3.5	0.0000
**APOBEC3G**	**60489**	2.6	0.0000	2.5	0.0000
**CIART**	**148523**	0.5	0.0000	0.6	0.0000
**ZBP1**	**81030**	1.8	0.0000	1.6	0.0011
**RARRES1**	**5918**	1.8	0.0027	1.6	0.0086
**TLR7**	**51284**	4.4	0.0000	3.1	0.0000
**THRA**	**7067**	0.5	0.0000	0.6	0.0016
**HLA-C**	**3107**	1.9	0.0000	2.0	0.0000
**MLKL**	**197259**	2.2	0.0000	1.9	0.0000
**CD80**	**941**	1.7	0.0016	1.7	0.0010
**CD163**	**9332**	1.9	0.0000	2.4	0.0000
**CXCL13**	**10563**	2.8	0.0000	1.8	0.0009
**AKT1**	**207**	1.6	0.0145	1.7	0.0010
**WNT16**	**51384**	0.5	0.0000	0.5	0.0000
**CCL8**	**6355**	2.3	0.0000	1.8	0.0063
**CYBB**	**1536**	3.3	0.0000	2.4	0.0000
**GZMK**	**3003**	2.5	0.0000	1.7	0.0010
**ITGA4**	**3676**	2.3	0.0000	1.9	0.0000
**IFI44L**	**10964**	23.3	0.0000	15.3	0.0000
**RGS1**	**5996**	4.3	0.0000	2.0	0.0000
**IFI44**	**10561**	20.5	0.0000	13.7	0.0000
**VCAM1**	**7412**	1.8	0.0000	1.6	0.0021
**CSK**	**1445**	1.7	0.0000	1.9	0.0000
**GBP1**	**2633**	7.8	0.0000	5.4	0.0000
**IRF8**	**3394**	2.3	0.0000	2.1	0.0000
**SELL**	**6402**	1.8	0.0013	1.5	0.0019
**ICOS**	**29851**	1.5	0.0129	1.5	0.0055

**CCL4**	**6351**	2.9	0.0000	2.4	0.0000
**BLNK**	**29760**	1.8	0.0000	1.6	0.0073
**CCR1**	**1230**	2.5	0.0000	2.3	0.0000
**S100A9**	**6280**	8.6	0.0000	7.8	0.0000
**STAT4**	**6775**	1.9	0.0000	1.5	0.0021
**MX2**	**4600**	5.0	0.0000	3.3	0.0000
**BIRC3**	**330**	3.8	0.0000	2.6	0.0000
**LCP2**	**3937**	3.0	0.0000	2.3	0.0000
**MMP1**	**4312**	2.3	0.0233	1.8	0.0327
**EPSTI1**	**94240**	6.6	0.0000	4.3	0.0000
**GZMB**	**3002**	4.4	0.0000	3.7	0.0000
**EGFR**	**1956**	0.6	0.0037	0.6	0.0254
**CLEC4E**	**26253**	1.8	0.0027	1.5	0.0062
**FRZB**	**2487**	0.6	0.0014	0.7	0.0056
**HCST**	**10870**	1.6	0.0000	1.6	0.0016
**CD2**	**914**	3.2	0.0000	2.4	0.0000
**CD83**	**9308**	1.7	0.0023	1.5	0.0078
**CGAS**	**115004**	2.0	0.0000	1.7	0.0000
**GBP2**	**2634**	1.9	0.0000	1.8	0.0011
**TNFSF13B**	**10673**	3.1	0.0000	2.1	0.0000
**GZMA**	**3001**	3.6	0.0000	2.4	0.0000
**PTPRC**	**5788**	4.8	0.0000	3.2	0.0000
**CD69**	**969**	3.4	0.0000	2.1	0.0000
**C1QB**	**713**	2.5	0.0000	2.3	0.0000
**PPARGC1A**	**10891**	0.4	0.0000	0.5	0.0009
**CD38**	**952**	1.9	0.0000	1.7	0.0019
**SLAMF6**	**114836**	2.2	0.0000	1.8	0.0000
**VAV1**	**7409**	1.5	0.0000	1.6	0.0009
**PYHIN1**	**149628**	2.7	0.0000	1.9	0.0000
**LYZ**	**4069**	4.3	0.0000	3.7	0.0000
**CCL3**	**6348**	1.7	0.0068	1.6	0.0176
**CCL19**	**6363**	2.1	0.0056	1.7	0.0185
**CXCL11**	**6373**	12.7	0.0000	6.8	0.0000
**CXCL9**	**4283**	22.1	0.0000	11.1	0.0000
**GATA3**	**2625**	0.5	0.0012	0.7	0.0254
**IKZF1**	**10320**	1.9	0.0000	1.9	0.0000
**CASP1**	**834**	2.3	0.0000	2.1	0.0000
**DUSP6**	**1848**	1.5	0.0007	1.6	0.0128
**CLEC7A**	**64581**	2.6	0.0000	2.6	0.0000
**IL7R**	**3575**	8.7	0.0000	4.7	0.0000
**RTP4**	**64108**	1.9	0.0000	1.6	0.0010
**ISG15**	**9636**	8.6	0.0000	7.5	0.0000

**TDP2**	**51567**	1.5	0.0027	1.6	0.0386
**PIAS3**	**10401**	0.6	0.0016	0.6	0.0000
**CAT**	**847**	0.6	0.0051	0.6	0.0021
**CD274**	**29126**	1.7	0.0051	1.7	0.0121
**CKS2**	**1164**	2.0	0.0007	1.6	0.0085
**BST2**	**684**	7.6	0.0000	4.6	0.0000
**NMI**	**9111**	3.3	0.0000	2.6	0.0000
**IFIT1**	**3434**	7.0	0.0000	4.7	0.0000
**EIF2AK2**	**5610**	4.5	0.0000	2.9	0.0000
**TNFSF10**	**8743**	3.2	0.0000	2.3	0.0000
**B2M**	**567**	2.7	0.0000	2.5	0.0000
**TLR6**	**10333**	1.7	0.0000	1.6	0.0045
**PARP9**	**83666**	3.8	0.0000	3.1	0.0000
**IFIH1**	**64135**	6.5	0.0000	4.4	0.0000
**ISG20**	**3669**	1.8	0.0008	1.5	0.0058
**AIF1**	**199**	1.6	0.0000	1.6	0.0010
**ERAP2**	**64167**	2.6	0.0000	1.9	0.0000
**PTEN**	**5728**	2.2	0.0000	1.9	0.0016
**STAT2**	**6773**	3.1	0.0000	2.4	0.0000
**CDH1**	**999**	0.6	0.0077	0.6	0.0099
**LEPR**	**3953**	0.6	0.0027	0.6	0.0111
**AIM2**	**9447**	3.8	0.0000	2.7	0.0000
**DTX3L**	**151636**	4.8	0.0000	3.5	0.0000
**IL10RA**	**3587**	1.7	0.0008	1.8	0.0009
**TAP1**	**6890**	1.7	0.0031	1.5	0.0063
**RSAD2**	**91543**	4.2	0.0000	3.2	0.0000
**SAMHD1**	**25939**	2.6	0.0000	2.2	0.0000
**OAS1**	**4938**	8.9	0.0000	6.1	0.0000
**DDX60**	**55601**	2.5	0.0000	2.0	0.0009
**IFIT2**	**3433**	5.0	0.0000	3.4	0.0000
**CD55**	**1604**	0.5	0.0042	0.6	0.0211
**IRF6**	**3664**	0.6	0.0000	0.7	0.0056
**NFIA**	**4774**	0.5	0.0000	0.5	0.0000
**CTSS**	**1520**	3.1	0.0000	2.5	0.0000
**IFI6**	**2537**	6.3	0.0000	5.0	0.0000
**TRIM22**	**10346**	3.5	0.0000	3.0	0.0000
**IFI16**	**3428**	3.0	0.0000	2.2	0.0000
**OASL**	**8638**	2.4	0.0000	2.3	0.0000
**MDM2**	**4193**	1.8	0.0018	1.6	0.0268
**FPR1**	**2357**	1.6	0.0007	1.7	0.0247
**LYN**	**4067**	2.2	0.0000	1.8	0.0009
**SPI1**	**6688**	1.9	0.0000	2.0	0.0000

**STAT1**	**6772**	8.1	0.0000	6.1	0.0000
**MX1**	**4599**	10.3	0.0000	6.9	0.0000
**IFI30**	**10437**	1.9	0.0000	1.9	0.0000
**PSMB9**	**5698**	1.7	0.0000	1.6	0.0010
**IFI27**	**3429**	3.3	0.0000	3.2	0.0000
**PTPN6**	**5777**	1.8	0.0000	1.6	0.0000
**DDX58**	**23586**	5.2	0.0000	3.7	0.0000
**E2F3**	**1871**	1.6	0.0000	1.7	0.0000
**CMPK2**	**129607**	5.0	0.0000	3.7	0.0000
**IRF9**	**10379**	1.7	0.0000	1.8	0.0000
**FADS2**	**9415**	0.4	0.0409	0.3	0.0366
**PARP14**	**54625**	7.1	0.0000	5.3	0.0000
**XAF1**	**54739**	4.1	0.0000	3.1	0.0000
**TLR3**	**7098**	1.9	0.0000	1.5	0.0176
**OAS2**	**4939**	5.2	0.0000	4.1	0.0000
**NFIB**	**4781**	0.6	0.0036	0.6	0.0159
**IRF7**	**3665**	1.6	0.0000	1.6	0.0010
**IFIT3**	**3437**	9.5	0.0000	5.9	0.0000
**SUB1**	**10923**	1.8	0.0117	1.9	0.0148
**VHL**	**7428**	1.5	0.0021	1.5	0.0085
**NLRC5**	**84166**	1.8	0.0000	1.7	0.0000
**BTN3A1**	**11119**	2.4	0.0000	2.0	0.0000
**NT5C3A**	**51251**	2.4	0.0000	2.0	0.0022
**PML**	**5371**	1.8	0.0000	1.8	0.0000
**USP18**	**11274**	4.3	0.0000	3.9	0.0000
**APOL1**	**8542**	4.4	0.0000	3.9	0.0000
**SAMD9**	**54809**	4.9	0.0000	2.9	0.0000
**IFITM3**	**10410**	1.6	0.0027	1.7	0.0022
**SLC20A1**	**6574**	1.7	0.0082	1.6	0.0084
**IFI35**	**3430**	1.7	0.0016	1.6	0.0116
**IFITM1**	**8519**	4.4	0.0000	3.5	0.0000
